# Mitochondrial DNA Depletion Syndrome and Its Associated Cardiac Disease

**DOI:** 10.3389/fcvm.2021.808115

**Published:** 2022-02-14

**Authors:** Haiying Wang, Yijun Han, Shenwei Li, Yunan Chen, Yafen Chen, Jing Wang, Yuqing Zhang, Yawen Zhang, Jingsuo Wang, Yong Xia, Jinxiang Yuan

**Affiliations:** ^1^Department of Physiology, Institute of Basic Medical College, Jining Medical University, Jining, China; ^2^Clinical Medical College, Jining Medical University, Jining, China; ^3^Institute of Basic Medical College, Jining Medical University, Jining, China; ^4^Dongying Fifth People's Hospital, Dongying, China; ^5^Key Laboratory of Precision Oncology of Shandong Higher Education, Institute of Precision Medicine, Jining Medical University, Jining, China; ^6^The Collaborative Innovation Center, Jining Medical University, Jining, China

**Keywords:** mitochondrial DNA depletion syndrome, nuclear gene mutation, mtDNA, cardiac disease, ATP

## Abstract

Mitochondria is a ubiquitous, energy-supplying (ATP-based) organelle found in nearly all eukaryotes. It acts as a “power plant” by producing ATP through oxidative phosphorylation, providing energy for the cell. The bioenergetic functions of mitochondria are regulated by nuclear genes (nDNA). Mitochondrial DNA (mtDNA) and respiratory enzymes lose normal structure and function when nuclear genes encoding the related mitochondrial factors are impaired, resulting in deficiency in energy production. Massive generation of reactive oxygen species and calcium overload are common causes of mitochondrial diseases. The mitochondrial depletion syndrome (MDS) is associated with the mutations of mitochondrial genes in the nucleus. It is a heterogeneous group of progressive disorders characterized by the low mtDNA copy number. *TK2, FBXL4, TYPM*, and *AGK* are genes known to be related to MDS. More recent studies identified new mutation loci associated with this disease. Herein, we first summarize the structure and function of mitochondria, and then discuss the characteristics of various types of MDS and its association with cardiac diseases.

## Structure and Function of Mitochondria

### Mitochondrial Structure

The mitochondrion is a double membraned organelle commonly found in eukaryotic cells. It consists of four compartments: an outer mitochondrial membrane (OMM), an inner mitochondrial membrane (IMM), a mitochondrial intermembrane space (IMS), and membrane matrix. The OMM is smooth and contains plenty of pore-forming components. The IMM curves inward and folds into a crest containing a large number of enzymes. It is the site of biological oxidation, a process involving respiratory chain complex (CI-IV) and ATP synthase (CV) (1). IMS, located between the OMM and IMM, contains cytochrome c (Cyt-c), ADP/ATP carrier (AAC), and biotic factors and enzyme proteins including apoptosis-inducing factor (AIF) and pro-caspase 2, 3, 9. Mitochondrial permeability transition pores (MPTP) exist at the OMM-IMM contact site. The opening of MPTPs is related to mitochondrial membrane permeability and is subjected to regulation by lipometabolism, mitochondrial membrane potential change, oxidative stress, and other factors (2). Membrane matrix is a single continuous space containing reactive enzymes, mitochondrial genome, mitochondrial DNA (mtDNA), ribosomal RNA (rRNA), and transfer RNA (tRNA), which have functional roles in biotransformation and synthesis. Mitochondria are semi-autonomous organelles that are strongly dependent on the biological function of nuclear genes. In mammals, mtDNA consists of 37 genes, including 22 tRNA genes, 13 genes encoding mitochondrial respiratory chain and oxidative phosphorylation-related protein subunits that assemble respiratory chain complex I-IV(CI-CIV), and 2 rRNA genes (12S and 16S) (3). The majority of mitochondrial proteins are encoded by nucleic genes which first synthesize precursor proteins, and then process in the mitochondria to form mature proteins (4). Mitochondria are the “power workshops” of cells. They provide energy for cellular activities and perform biological functions such as signal transduction and biological oxidation.

### Mitochondrial Function

#### ATP Production by Oxidative Phosphorylation

**Figure 1:**
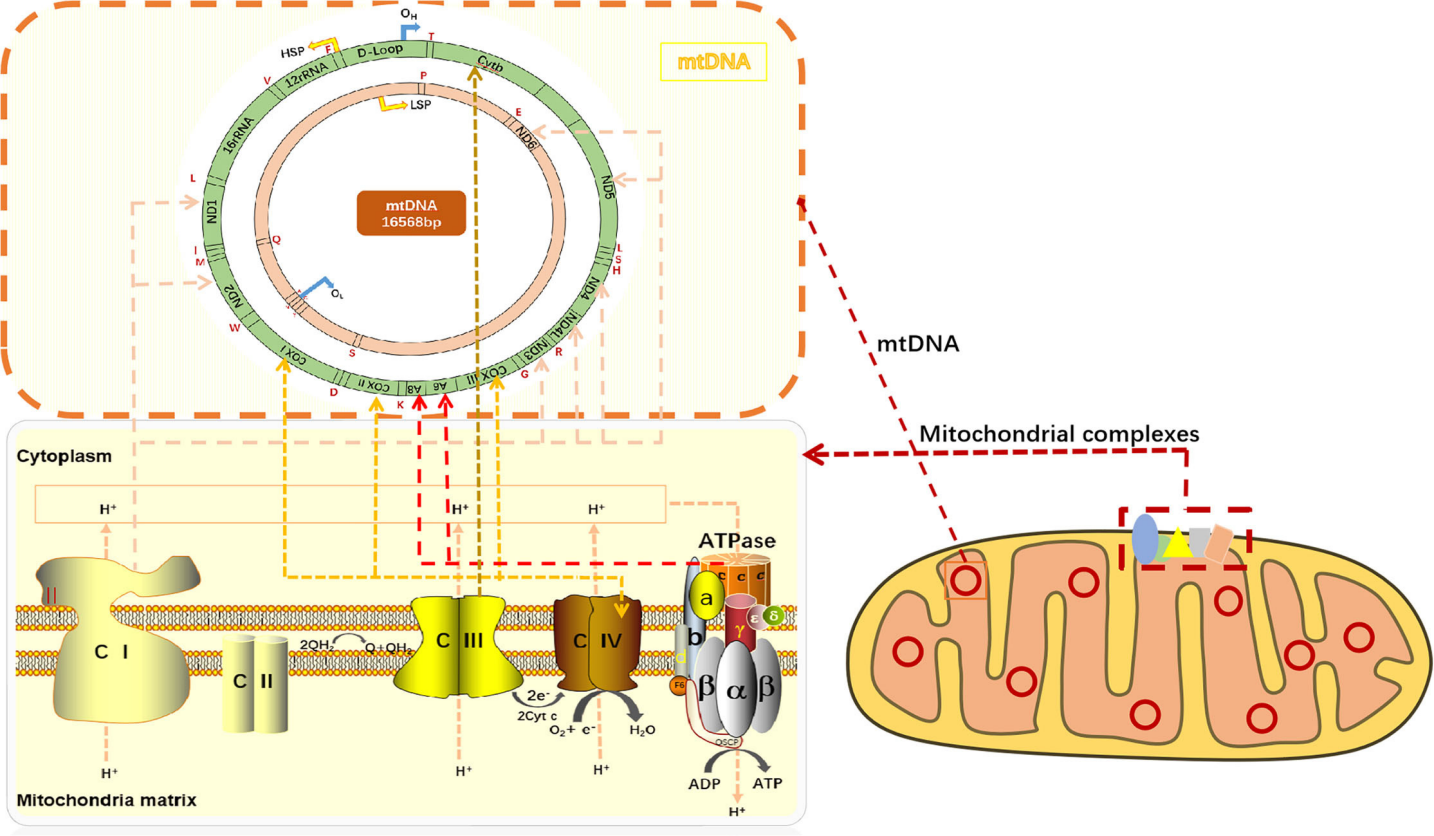
mtDNA encodes respiratory chain complexes for electron transport. Mitochondrial respiratory chain complex I (NADH oxidase) and mitochondrial respiratory chain complex II (succinate dehydrogenase) are the major components of electron entry into mitochondrial electron transport chain (ETC). Complexes I and II catalyze NADH oxidation and succinic acid oxidation to fumaric acid, respectively. Next, coenzyme Q (Q) forms coenzyme (QH2), leading to a decrease in oxygen (the final electron receptor). Mitochondrial respiratory chain complex III (cytochrome c oxidoreductase) is the gatekeeper of the mitochondrial respiratory chain and the main source of reactive oxygen species III, which transfers electrons from panthepanol (reduced coenzyme Q or CoQ) to cytochrome c (cyt c) through a catalytic mechanism of the Q cycle. The mitochondrial respiratory chain complex IV (cytochrome c oxidase) is the final electron acceptor of the mitochondrial ETC, which converts oxygen into water through the oxidation of cyt c, a process associated with ATP synthesis in the mitochondrial membrane. Mitochondrial respiratory chain complex V, also known as F1F0-ATPASE or ATP synthase, works with the four complexes to complete oxidative phosphorylation for ATP production. CI or NADH: Ubiquinone oxidoreductase or NADH encoded by ND1, ND2, ND3, ND4, ND4L, ND5, and ND6 on mtDNA, respectively; CII: dehydrogenase succinic acid, ubiquinone oxidoreductase; CIII: Ubiquinone and ferricytochrome C reducing enzyme encoded by Cytb part on mtDNA, respectively; CIV: CIV, cytochrome c oxidoreductase encoded by COXI, COXII, and COXIII on mtDNA, respectively; The red letters T, F, V, L, I, M, W, D, K, G, R, H, S, L, A, N, C, Y, S, E, P, and Q all indicate tRNA.

Mitochondria is the metabolic center of cellular activities. ATP is often called “energy currency” of the cell, and the breakage of high energy phosphate bonds by hydrolysis can provide energy for cellular activities. ATP synthesis is indispensable of oxidative phosphorylation (OXPHOS). OXPHOS is closely related to material energy metabolism (5), electron transfer of the mitochondrial respiratory chain and the enzymatic reaction of a series of protease complexes. OXPHOS contributes to ninety percent of the energy production in eukaryotic cells. On the lipid double layers embedded in IMM, electrons are transmitted to O_2_ by compound I-IV on the respiratory chain, while simultaneously releasing energy for ATP production in coupling with F0/F1-ATP synthase (complex V) (6) (Figure 1). ATP synthesis is impaired when mitochondrial phosphorylation function becomes dysregulated.

##### Changes in the Mitochondrial Membrane Potential (Δψ) via Electron Transfer in the Mitochondrial Respiratory Chain

The transmission of mitochondrial respiratory chain is indispensable of electronic transfer. Therefore, the process is known as the electron transport chain (ETC). Mitochondria pass through complexes I (CI or NADH: ubiquinone oxidoreductase or NADH dehydrogenase), II (CII, succinic acid: ubiquinone oxidoreductase), III (CIII, ubiquinone: Ferric cytochrome C reductase) and IV (CIV, cytochrome C oxidoreductase), transfer electrons to O_2_, and release energy which couples with complex V (CV, ATP synthase) to form ATP (7). In mammals, CI is the largest mitochondrial complex composed of 45 subunits, of which 7 are encoded by mitochondrial DNA, namely ND1, ND2, ND3, ND4, ND4L, ND5, and ND6, and the remaining 38 subunits are encoded by nuclear genes. NADH: ubiquinone oxidoreductase subunit AB1 (NDUFAB1), or known as mitochondrial acyl carrier protein, is an important subunit of CI, which can mediate cardioprotective mechanisms and improve mitochondrial bioenergy (8). CIV consists of 13 subunits, of which the largest three subunits (COX I, COX II and COX III) are encoded by mtDNA and can form the core of the cascade reaction, while the remaining 10 subunits (COIV, Va, Vb, VIa-c, VIIa-c, and VIII) are encoded by nDNA. Moreover, the activity of CIV is highly relevant to muscle OXPHOS, followed by CII. CIIIis the most critical mitochondrial respiratory chain enzyme, consisting of 11 different subunits, of which only Cytb is encoded by mtDNA, and Cytb forms a dimer with Cyt C_1_ and iron-sulfur protein (Fe-S) (9). CIII transfers electrons from panthenol (reductive coenzyme Q or CoQ) to cytochrome C (Cyt C) through a catalytic mechanism, while protons are transferred from the mitochondrial matrix to the IMS (10) (Figure 1). In the process of electron transfer, the complexes interact with each other to form an advanced supramolecular structure known as the supercomplex (SCs) and respiratory corules (11). SCs can improve the efficiency of electron flux through substrate channels or enhanced catalysis, thus promoting electron transport, isolating reaction intermediates and preventing reactive oxygen species (ROS) generation (12).

##### Mitochondrial and Reactive Oxygen Species

During ATP generation, ETC, and Krebs cycle inevitably lead to electron leakage, causing incomplete reduction of O_2_ and formation of free radicals in a form of superoxide anion (O^2−^). At this point, if the muscle remains in a long-term contraction state, the content of ATP generated by oxidative phosphorylation of respiratory chain increases, and electrons overflow into ETC to generate ROS (13). Furthermore, a low level of ROS can act as signaling molecules that maintain normal cell signal transduction. In contrast, a high level of ROS produced more than endogenous scavenging capacity can trigger cascade reactions that damage cellular macromolecules, such as DNA and proteins. Excessive ROS can also reduce the Ca^2+^sensitivity of myofibril and damage the contractile function of muscle, possibly through enzyme complexes i and iii (Ci, Ciii)-dependent and/or reduced nicotinamide adenine dinucleotide (NADPH) oxidase-dependent pathways. Alternatively, α-ketoglutaric acid in succinyl-coenzyme A can be oxidized indirectly through mitochondria dehydrogenase to generate NADH, which is responsible for ROS overproduction (14). It has been shown that ROS produced by ETC can damage mtDNA. In the absence of proper repair, DNA damage accumulates, causing a series of mitochondrial-related diseases (15).

#### Role of Oxygen in Mitochondrial Function

Oxygen is key to the functionality of mitochondria. Oxygen affects normal structure and function of the mitochondria. In the third stage of aerobic respiration, mitochondria use NADH, a product of glycolysis and Tricarboxylic Acid Cycle (TCA), reduced flavin adenosine dinucleotide, FADH_2_ and other high-energy molecules to reduce oxygen, thereby releasing energy to synthesize ATP. The content of oxygen in the environment is closely related to normal oxidative phosphorylation function of mitochondria (16, 17). In mammals, hypoxic signaling protein (HIF) determines the cellular response to hypoxia. Stable HIF protein controls the expression of glycolysis-related target genes at an appropriate level and maintains ROS production at a low, physiological level by changing the structure of CI, II and IV ETC complexes and reducing the enzyme activity of the complex (18). The morphology of fetal cardiac mitochondria at birth is associated with hypoxia signaling. Downregulation of mitochondrial HIF pathway after birth leads to increased respiratory capacity and oxidative phosphorylation. Neary et al. found that HIF signal transduction was maintained under normoxic conditions in mouse heart. The α-subunit proline residues of HIF-α are continuously hydroxylated by Prolyl Hydroxylase (PHD) under normoxic conditions. Hypoxia inhibits PHD, which stabilizes HIF-α and promotes its migration to the nucleus where it dimers with the β subunit and binds to the promoters or enhancers through the hypoxia response elements (HRE) to initiate gene transcription (19, 20). Mitochondria are the main oxygen consumers in the heart. When cardiomyocytes are hypoxic, anaerobic glycolysis increases whereas both mitochondrial oxygen consumption and respiration rate decrease. Ambrose et al. have established a cell model of cardiac hypoxia by using HL-1 cardiomyocytes cells highly related to adult cardiac metabolism. The investigators found that the number of mitochondria increased with a simultaneous decrease in CIV activity after 24 h of hypoxia, suggesting that mitochondria keep their total content fairly consistent during remodeling (21).

#### Mitochondria and Cell Metabolism

Mitochondria achieve metabolic functions mainly by oxidative phosphorylation, ATP production, NADH oxidation, glycolysis and FADH_2_ production, TCA cycle, and β–oxidation of fatty acids (22). Meanwhile, mitochondria regulate their fusion, fission, biosynthesis, and degradation through metabolism.

##### Metabolism-Related Biochemical Reactions

Metabolism is fundamental to cellular activities. It represents distinct processes by which human body converts the nutrients, including sugars, lipids and proteins, into small molecules for absorption and consumption. Fatty acid β -oxidation (FAO) is a major process of long-chain fatty acid metabolism. It produces acetyl coenzyme A (acetyl-CoA), NADH, and FADH_2_ (23). The TCA, also known as the Krebs cycle, is the hub that links the metabolism of three nutrients. Acetyl-CoA is a common end product of nutrient metabolism, which eventually enters TCA in the form of citrate for thorough oxidation (24). Acetyl CoA is decomposed into CO_2_ and H. H is delivered to flavin adenine dinucleotide (FAD) and oxidized nicotinamide adenine dinucleotide (NAD+) to generate FADH_2_ and NADH (25). Metabolism and energy homeostasis are maintained through the activities of various enzymes including AMP-activated protein kinase (AMPK), a conserved protein present in all eukaryotes. AMPK is involved in the metabolism of lipids and carbohydrates. AMPK is activated when the intracellular AMP concentration increases or the skeletal muscle contracts. The activity of AMPK is also regulated by hormones, cytokines, and ROS (26).

##### Metabolic Regulation of Mitochondrial Dynamics

Mitochondria are highly dynamic organelles. Mitochondrial dynamics is a physiological process in which mitochondria undergo continuous cycles of fusion and division, resulting in changes in the shape, size and position of mitochondria. Mitochondrial dynamics is often determined by the dynamic balances between fission and fusion as well as between biogenesis and degradation such as mitochondrial phagocytosis (27). Mitochondrial fission and fusion are of great importance in the growth and redistribution of mitochondria and the maintenance of a healthy mitochondrial network (28, 29). Through continuous fusion and fission, mitochondria can exchange the damaged mitochondrial DNA and protein lipids with normal counterparts, thus maintaining the stability of mitochondrial structure and function (30). During this process, the core proteins that mediate mitochondrial division and fusion are membrane remodeling mechanochemical enzymes, such as dynein-related protein-like 1 (Drp1/DLP1), optic nerve atrophy 1 (OPA1), and mitochondrial protein (Mfn). Drp1 regulate fission, while OPA1 and Mfn modulate the fusion of IMM and OMM, respectively (31). It is likely that the processes of mitochondrial fission and fusion are asymmetric in the formation of daughter mitochondria. It has been found that the dynamic changes in mitochondria and CR structure formed by endointimal folding have a significant impact on the integrity of mtDNA. CR distortion often leads to mtDNA loss (32). Genetic loss of mitochondrial fusion can result in inadequate mitochondrial mixing, which links to great heterogeneity of mitochondrial proteins, DNA, and membrane potentials. A function of fission is to identify dysfunctional offspring mitochondria, which are subsequently removed by mitochondrial phagocytosis and coordinate for inhibition of mitochondrial fusion (33). Normal mitochondrial biogenesis depends not only on the action of nDNA and mtDNA, but also on the coordination of mitochondrial DNA replication and fusion with division. Environmental factors, such as oxidative stress, exercise, temperature stress (cold or heat), cell division, regeneration and differentiation, can promote mitochondrial biogenesis. Inhibition of mitochondrial dynamics has some effects on the elimination of irreparable mtDNA damage and transmission of mtDNA mutations (34).

#### Ca^2+^ Storage

Accumulation of Ca^2+^ in the mitochondrial matrix regulates aerobic metabolism by activating enzymes in the metabolic reactions. Under physiological conditions, Ca^2+^ actively regulates the key dehydrogenase in the TCA response, which is conducive to the regulation of Ca^2+^-dependent functions in the cytoplasm. Under pathological conditions, high Ca^2+^ concentration can promote the opening of mitochondrial permeability transition pore (MPTP) and induce apoptotic death (35, 36). Excessive deposition of Ca^2+^ often leads to decreased ATP synthesis and increased ROS production, thus aggravating oxidative stress responses. Ca^2+^ overload is often associated with intracellular Ca^2+^ leakage. Sarcoplasmic reticulum (SR) Ca^2+^ leakage mainly occurs through type 2 Ryanodine receptor (RyR2). It plays a fundamental role in Ca^2+^ pathophysiology. RyR2 regulates the level of Ca^2+^ by influencing cardiac excitation-contraction (E-C) coupling (37). Ca^2+^ concentration in mitochondria is also regulated by genes encoding Ca^2+^ unidirectional complexes, including mitochondrial calcium uniporter (MCU) and its regulator Ca^2+^ uptake protein 1 located in the IMM (38). Ca^2+^ absorption is dependent on the energy transfer of the respiratory chain. Due to the restriction of IMM on MCU, a high level of Ca^2+^ is required for MCU to enter the mitochondrial matrix, thus, Ca^2+^ absorption is relatively easy in the vicinity of Ca^2+^ release reservoirs, such as the endoplasmic reticulum (ER). Mitochondria-associated endoplasmic reticulum membrane (MAM) has been found to transport Ca^2+^. Ca^2+^ can be directly transferred to mitochondria *via* ER membrane where it controls the relevant mitochondrial functions (39).

#### Mitochondrial Genome Regulation of Its Function

The mitochondrial genome consists of 37 genes, including 22 tRNA genes, 13 genes that encode the subunits of mitochondrial respiratory chain and oxidative phosphorylation-related proteins snd 2 rRNA genes (12S and 16S). Nuclear genes encode about 1,500 mitochondrial proteins, which comprise the majority of mitochondrial respiratory chain polypeptides (40). Additionally, mtDNA also encodes 13 core catalytic peptides, including oxidative phosphorylation complexes i, iii, and iv (41).

It appears that mtDNA depends on mitochondrial nDNA to complete its replication, transcription, translation as well as other processes. Therefore, both nDNA and mtDNA have the regulatory effects on mitochondria, and they jointly ensure the normal function of the mitochondria (42). Given that mtDNA encodes subunits of the respiratory chain complex, mutations in mtDNA can damage the enzymatic activity of oxidative phosphorylation reaction complex, in particular CI, affecting mitochondrial biogenesis (43). The level of mtDNA correlates to mitochondrial fusion. In skeletal muscle, the association between fusion and enzymatic activity in OXPHOS may limit mtDNA defects to the local regions of muscle fibers, which are often associated with mitochondrial myopathy (44). Altogether, mitochondria regulate cell metabolism, growth, senescence, and apoptosis through the coordinated actions of nDNA and mtDNA.

## Mitochondrial DNA Depletion Syndrome

As an autosomal recessive disease, mitochondrial DNA depletion syndrome (MDS) is characterized by severe reduction in mtDNA content in the affected tissues and organs (45). Because mtDNA is controlled by nDNA, the copy number of mitochondria may be reduced when the nuclear gene mutations destroy deoxyribonucleotide metabolism in the affected tissues. The production of key subunits of mitochondrial respiratory chain complex and energy production require a sufficient amount of mtDNA. Deletion of mtDNA can lead to organ dysfunction as a result of insufficient synthesis of respiratory chain components (46–48). Commonly mutated genes include *TK2, FBXL4, TYPM, AGK*, and others. MDS is classified as four forms, which are myopathic form, encephalomyopathic form, hepatocerebral form, and neurogastrointestinal form (49). MDS is usually involved in multiple systems with muscle-related symptoms, such as dystonia and muscle atrophy (50, 51).

### Myopathic Mitochondrial DNA Depletion Syndrome

Myopathic MDS, also known as mitochondrial DNA depletion syndrome 2 (MTDPS2) or *TK2* deficiency, is characterized by the reduction of mtDNA content in the affected tissues. It is often associated with severe progressive muscle weakness. Dysphagia mainly involves the respiratory system with other clinical manifestations (52). Some patients have clinical symptoms of hypertrophic cardiomyopathy (53). This type of MDS is usually caused by mutation of *TK2* gene. It occurs in early childhood and presents in the form of myopathy, mostly accompanied by mtDNA deletion/depletion and increased serum level of creatine kinase (CK) (54, 55). *TK2*, located on chromosome 16Q21, encodes a mitochondrial enzyme, thymidine kinase 2 (*TK2*). *TK2* is a rate-limiting enzyme which mediates deoxypyrimidine phosphorylation (56). In non-replicating cells, *TK2* is also involved in the maintenance of dNTP (57). In clinical and molecular genetics, myopathic MDS can be further divided into three different subtypes, namely, paroxysmal myopathy in infants with severe depletion of mtDNA, paroxysmal myopathy in childhood, and delayed myopathy, among which delayed myopathy is the rarest. In addition to muscle weakness, delayed myopathy can also progress to respiratory insufficiency. Different from the previous two, in addition to muscle weakness, respiratory insufficiency can also be developed at the onset and after 12 years old. Muscle mtDNA can show myopathy with deletion, multiple deletion, or both (58).

### Encephalomyopathic Mitochondrial DNA Depletion Syndrome

Encephalomyopathic is a relatively common form of MDS, in particular among adults who have progressive external eye muscle paralysis (adPEO). adPEO manifests by ptosis and severe limitation of eye movement. It may also lead to ataxia and cataract (59). Mitochondrial DNA depletion syndrome 13 (MTDPS 13), also known as *FBXL4*-related encephalopathy, is a form of MDS manifested by early lactic acidosis, hypotonia, developmental delay, and feeding difficulty (60). In some cases, encephalopathic MDS accompanies liver disease to form hepatocerebral MDS (59). Mitochondrial DNA depletion syndrome 9 (MTDPS 9) can also occur in patients with cerebromyopathic MDS. Lu et al. reported a case of infantile encephalomyopathic MDS associated with *SUCLG 1* (succinic acid A ligase α subunit) gene in 2017. The case is manifested by neuromyopathy-like symptoms including muscular hypotonia, psychomotor retardation, brain atrophy, developmental delay, and increased levels of methylmalonic acid and its metabolite (61). Encephalomyopathy can be caused by single gene or polygenic mutations with similar effects. These genes may have synergistic effect on the development of MDS (45).

### Hepatocerebral Mitochondrial DNA Depletion Syndrome

Hepatocerebral MDS is one of the most common MDS diseases, and at least 50 kinds of related gene mutations have been discovered (62). This disease may occur in newborns within 6 months after birth, as characterized by vomiting, developmental delay, serious progressive liver failure, hypotonia, hyperreflexia, irritability, and hypoglycemia. Patients often die within a year after the onset of symptoms (63). Hyperlactatemia and 3-methylglutaric aciduria are the well-known characteristics of patients with hepatic encephalopathy, while some patients are accompanied by nephrolithiasis or mild ventricular hypertrophy (64). The liver biopsy can show varying degrees of steatosis, bile duct hyperplasia, fibrosis, lobular collapse, and other symptoms. Studies have shown that most patients presented a pattern of fibrosis with neovascularization following diffuse necrosis (65). Reduction of cytochrome-C oxidase (COX) and combined defects of mitochondrial respiratory chain complex may also be detected in infants' livers (66). Clinically, the identified liver-brain type mutations include *POLG1, DGUOK, C10orf2*, and *MPV17*, among which *MPV17* is relatively rare (67).

### Neurogastrointestinal Mitochondrial DNA Depletion Syndrome

Mitochondrial neurogastrointestinal mitochondrial DNA depletion disease (MNGIE) is a rare metabolic disease caused by *TYMP* gene mutation encodes thymidine phosphorylase (TP). Patients with inherited, autosomal recessive *TYMP* mutations show clinical symptoms between 10 and 50 years old, most of which appear before 20 years old (68). In MNGIE, a loss of TP activity leads to the accumulation of deoxyuridine (dUrd) and thymidine (Thd), which infiltrate through the mitochondrial pyrimidine remedy pathway, leading to an imbalance of deoxyuridine triphosphate pool (dNTP). This damages mtDNA, resulting in mitochondrial depletion and ultimately cell death (69). The clinical manifestations of MNGIE include gastrointestinal motility disorders, cachexia, ptosis, ophthalmoplegia, peripheral neuropathy, and related encephalopathy, which often lead to death in adulthood (70). The detection of hypoxia inducible factor-1α (HIF-1α) and vascular endothelial growth factor (VEGF) protein expression proved that MNGIE patients were accompanied by gastrointestinal vascular diseases (71). Currently, hemodialysis can temporarily eliminate the toxic effects of Thd and dUrd in patients. However, permanent TP replacement therapy with AHSCT is required for more effective treatments of MNGIE (69). The correlation between MNGIE vascular changes and neuromuscular abnormalities provides a new strategy for the treatment of this disease (72). Carrier erythrocyte entrapped thymidine phosphorylase therapy and allogeneic hematopoietic stem cell transplantation are currently available clinical therapeutic options for MNGIE (71).

## MDS and Its Association With Cardiac Disease

Highly energy-consuming organs including heart requires a proper function of mitochondria. Cardiac energy is primarily provided through mitochondrial OXPHOS (73). Cardiomyocytes are prone to hypoxia. There are two mitochondrial populations in cardiomyocytes, namely, interfibrous mitochondria and submyomembrane mitochondria. The interstitial mitochondria are arranged longitudinally and in parallel. The cristae are mainly arranged in tubular shape. Subsarcolemmal mitochondria gather beneath the sarcolemma in the direction of lamellar ridge (74). Mitochondria are vital for the development and maintenance of heart muscle, the largest energy user in the human body. Mitochondria are not only a source of ATP energy, but also generators of ROS, which can both regulate physiological processes, such as the transition from hyperplastic growth to hypertrophic growth after birth, and cause oxidative damage. Excessive ROS production and oxidative damage are associated with cardiac pathology (75). In addition to lowering bioenergy efficiency, damaged mitochondria also produce more reactive oxygen species, resulting in harmful functional and structural consequences in the cardiovascular system (76). The increased production of ROS leads to vascular inflammation, endothelial cell damage and deposition of oxidized low density protein (oxLDL) in the arterial wall (77). In cardiomyocytes, two mitochondrial populations are electrically coupled to each other, providing electrical conduction from one to the other (78). Dysfunctional mitochondria can impair electronic transfer chain activity, cause ATP depletion, increase oxidative stress, and activate intrinsic apoptosis, leading to myocardial cell death (79). Metabolism is not only crucial for regulating the pump function of the heart, but also for maintaining the functional balance and cellular senescence of cardiomyocytes (80). The important functions of mitochondria in redox balance, energy metabolism and calcium balance determine its pathogenic role in heart failure (HF) and cardiovascular disease (CVD) (81–83). There is no effective treatment at present (84). Accumulation of evidence on mitochondrial therapy may help to provides new targets for the treatment of MDS-associated heart disease (85).

Mitochondria, as an important energy “power plant” of the human body, are closely related to the function of the heart. The changes in mitochondrial structure and physiological function play important roles in heart development, heart remodeling, heart failure, and ischemia-reperfusion injury (86). Some cardiac diseases, such as cardiomyopathy, are mostly related to mitochondrial dysfunction. Pulmonary hypertension may lead to enlargement of the right ventricle. Persistent pulmonary hypertension of neonatal apical hypertrophic cardiomyopathy has been proven to be a rare manifestation related to mitochondrial dysfunction (87). When mitochondria are dysfunctional, mtDNA is damaged due to an increase in ROS production (88). Studies have found that MDS involves not only the brain, liver, skeletal muscle and other organs, but also the heart. The impaired mitochondrial DNA replication may result in mtDNA depletion, and ultimately leads to heart failure. Accordingly, the number of the protein encoded by mtDNA is also reduced (73). Santorelli et al. found a tissue-specific deletion of cardiac mtDNA in an infant patient with hypertrophic cardiomyopathy and mitochondrial exhaustion syndrome. There is increasing evidence that MDS can cause cardiomyopathy (89). In addition to the increased levels of blood lactic acid and pyruvate, liver injury and abnormal myocardial enzymes are also common in laboratory examination (90). Two out of twelve MDS children were reported to have elevated myocardial enzymes (91).

*FBXL4, ANT1, AGK*, and *SLC25A4* have been confirmed to be associated with cardiomyopathy. Wang et al. reported two cases of *AGK* mutation as a serious manifestation in infants with heart failure. Their skeletal muscle biopsy showed severe mtDNA depletion, along with muscle weakness, growth retardation, and other manifestations of muscle disorders. The cases were diagnosed as Sengers syndrome, thus providing strong evidence for the relationship between mitochondrial depletion syndrome and cardiomyopathy (92). Mitochondrial DNA mutation is considered to change the adaptation between bioenergy systems and dietary patterns, affect the cellular response to stimuli, and increase the susceptibility of mutant individuals to cardiometabolic diseases (93–96).

### MDS-Related Genes and Their Defects

#### TK2

*TK2* is a 16q21 gene located on chromosome 16, which encodes for thymidine kinase 2. Many *TK2* gene mutations are associated with MDS. Yan et al. demonstrated a mitochondrial DNA depletion syndrome-2 patient caused by *TK2* compound heterozygous mutation. The *TK2* mutation is considered a recessive genetic mutation type (97). In a case series of 18 patients, Cristina Domínguez-González et al. (99) found that nearly 80% of the patients developed MDS before the age of 12, and only 19% of the patients developed after the age of 12. Clinically, *TK2*-related MDS patients share common symptoms, including progressive myopathy, skeletal muscle involvement (e.g., facial and axial neck flexor weakness), respiratory system involvement, and chronic progressive external ophthalmoplegia (CPEO) (98). A previous study found that mtDNA depletion mostly affected the brain and heart tissues of *TK2* H126N knock-in mice (99). Measurements of mitochondrial *TK2* protein by immunoprecipitation found high levels of *TK2* in heart, liver, and spleen. The level of thymidylate synthase (TS) protein was also high in the heart tissue (100). Thymidine Triphosphate (TTP) is synthesized mainly by *TK2* in the heart. Kamath et al. found that thymidine monophosphate (TMP) could promote the synthesis of TTP independent of *TK2* in the cardiac mitochondria of rats *in vitro*, suggesting a potential therapeutic strategy for *TK2* deficiency-related diseases (101).

#### SUCLG1

**Figure 2:**
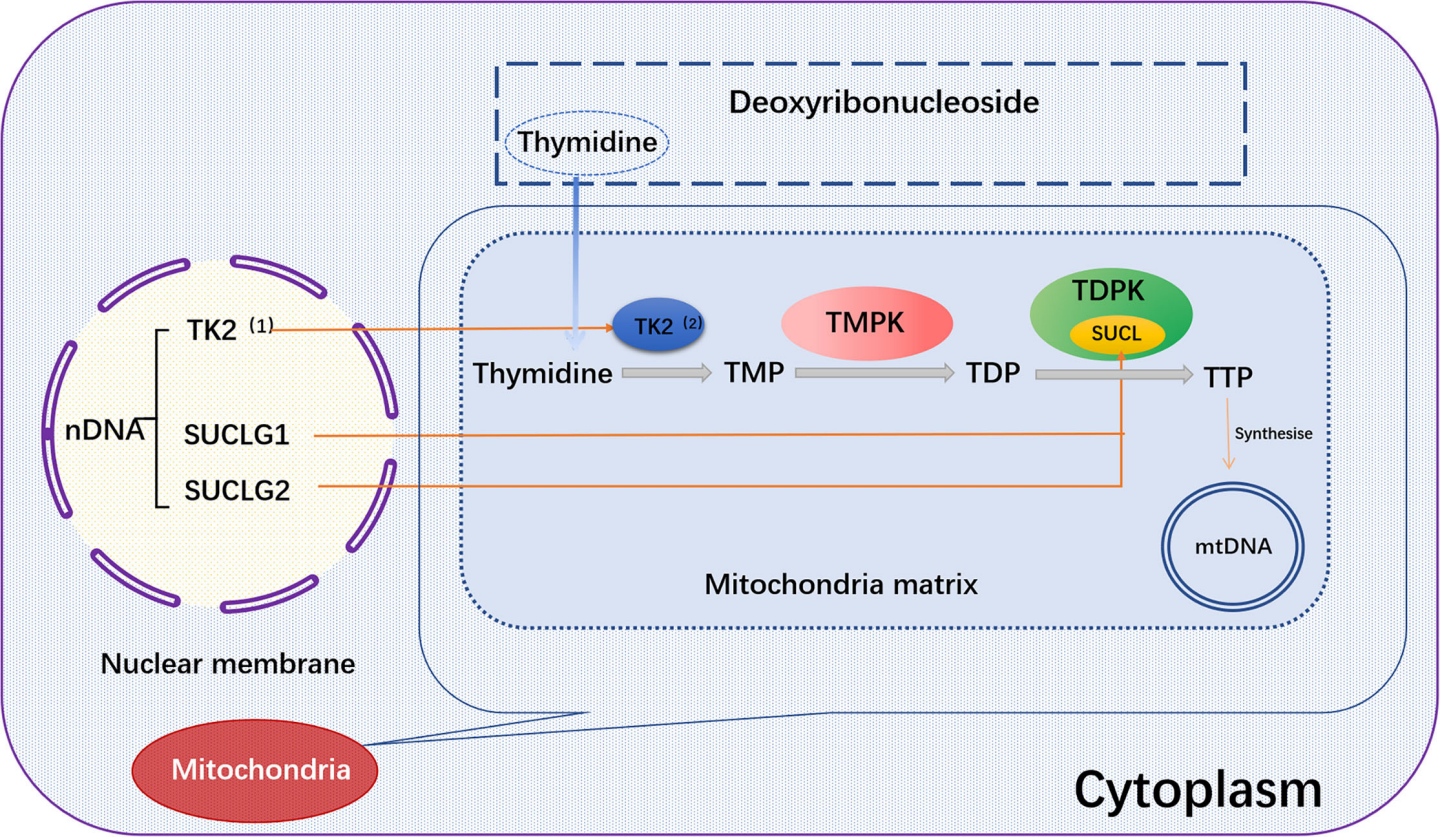
*TK2, SUCLG1*, or SUCLG2 regulates mtDNA synthesis. In the salvage pathway, the pre-formed deoxyribothymidine enters the mitochondria. The deoxyribonucleoside is then catalyzed by the thymokinases TMPK and TDPK to phosphorylate the deoxyribonucleoside into the triphosphate form in the subsequent three phosphorylation steps, and further synthesize mtDNA. Among them, *TK2* and TDPK are encoded by the nuclear genes *TK2* and *SUCLG1*/*SUCLG2*, respectively. *TK2*, (1) Thymidine Kinase 2, (2) Thymidine kinase; *SUCLG1*, succinic acid A ligase α subunit; SUCLG2, succinate-CoA ligase GDP-forming β subunit; Thymidine, deoxyribosylthymine; TMPK, Thymidylate kinase; TDPK, Thiamine diphosphokinase; SUCL, Succinyl CoA ligase; TMP, Thymidine monophosphate; TDP, Thymidine diphosphate; TTP, Thymidine 5'-triphosphate.

*SUCLG1* gene encodes the α-subunit of succinate COA ligases (SUCL, also known as succinyl CoA synthase). *SUCLG1* subunit is widely expressed in human tissues and abundant in the brain, heart, liver and kidney. A double allele mutation in *SUCLG1* gene can promote the deficiency of succinate synthase or ligase activity, resulting in the reduction of mtDNA synthesis and mtDNA depletion (102–104). Several studies have shown that *SUCLG1* mutations may be related to mitochondrial hepatic encephalopathy. Hove et al. reported that a neonatal patient had elevated transaminase and liver failure symptoms within 2 months after birth. The mtDNA content of the patient was also decreased, accompanied by progressive myopathies. Gene sequence analysis demonstrated a homozygous mutation [c.40A>T (p.M14L)] in *SUCLG1* gene (105). Clinical data showed that succinate ligase deficiency was associated with mtDNA depletion. Liu and co-workers reported five new *SUCLG1* mutations, including exon 5 c.550G >A (p.G184S), exon 7 C.751C>T (p.G251S), exon 7 c.809A>C (p.L270W), exon 8 c.961C>G (p.A321P), and exon 9 826-2A>G (splicing), in three Chinese patients with succinate-CoA ligase deficiency (103). The latter two mutations are speculated to be destructive. There are only few case reports linking *SUCLG1* mutation to heart diseases (106). Therefore, it is worth of tracking and evaluating the potential relationship between *SUCLG1* mutation and impairment of cardiac function due to the abundance of *SUCLG1* in the heart (Figure 2).

#### SLC25A4

*SLC25A4* gene is located on chromosome 4 and encodes for adenine nucleotide translocase 1 (ANT1), which is widely expressed in the heart and skeletal muscles. ANT1 translocates ADP/ATP across the IMM, provides ADP to the mitochondrial ATPase (Complex V), and plays an important role in ATP/ADP exchange between the cytosol and mitochondrial compartment (107, 108). Many studies have shown that *SLC25A4* is related to mtDNA depletion sign 12. Thompson et al. found that significant indigenous depletion of mtDNA caused severe mitochondrial respiratory chain deficiencies (109). They identified 2 pathogenic mutation sites: c.703C>G (p.Arg235Gly) and c.239G>A (p.Arg80His). *SLC25A4* c.239G>A was found in a case of mitochondrial exhaustion syndrome (MTDPS12) with epileptic encephalopathy. The patient was admitted to hospital due to continuous facial and limb spasm, accompanied by mild respiratory failure (110). A frame-shift null mutation (c.523delC, p.Q175RfsX38) of *SLC25A4* resulted in autosomal recessive myopathy and cardiomyopathy. Ten patients with homozygous mutation (adenine nucleotide transposon −1^−/−^) were followed up for 6 years. These patients displayed continuous adrenergic activation, exercise intolerance, lactic acidosis, hyperalaninemia, and progressive myocardial thickening. Moreover, ECG, echocardiography, and velocity vector imaging analyses indicated the dysregulated left ventricular relaxation, abnormal myocardial repolarization, and abnormal systolic mechanics. Extensive degeneration of cardiomyocytes and structural abnormalities of mitochondria were also evidence in these patients (111). The characteristics of myopathy and cardiomyopathy were also observed in mice lacking the heart/muscle isoform of ANT1 (112).

#### AGK

Sengers syndrome is an autosomal recessive genetic disease caused by *AGK* mutations. *AGK*, a mitochondrial membrane kinase, is involved in protein biogenesis (112). Besides, intracellular LPA is generated by the phosphorylation of monoacylglycerol by *AGK* (113). Vukotic et al. found that *AGK* belonged to a subunit of the TIM22 complex that facilitates the transport of metabolite carriers into the IMM. ANT1 levels were reduced in the cells of Sengers syndrome patients, suggesting that *AGK* is needed for the efficient insertion of metabolite carriers (e.g., SLC25A24 and ANT1) (114). The main clinical features of Sengers syndrome are hypertrophic cardiomyopathy, bi-sided cataracts, myopathy, and lactic acidosis. Hypertrophic cardiomyopathy can eventually contribute to the development of heart failure (91).

#### MPV17

**Figure 3:**
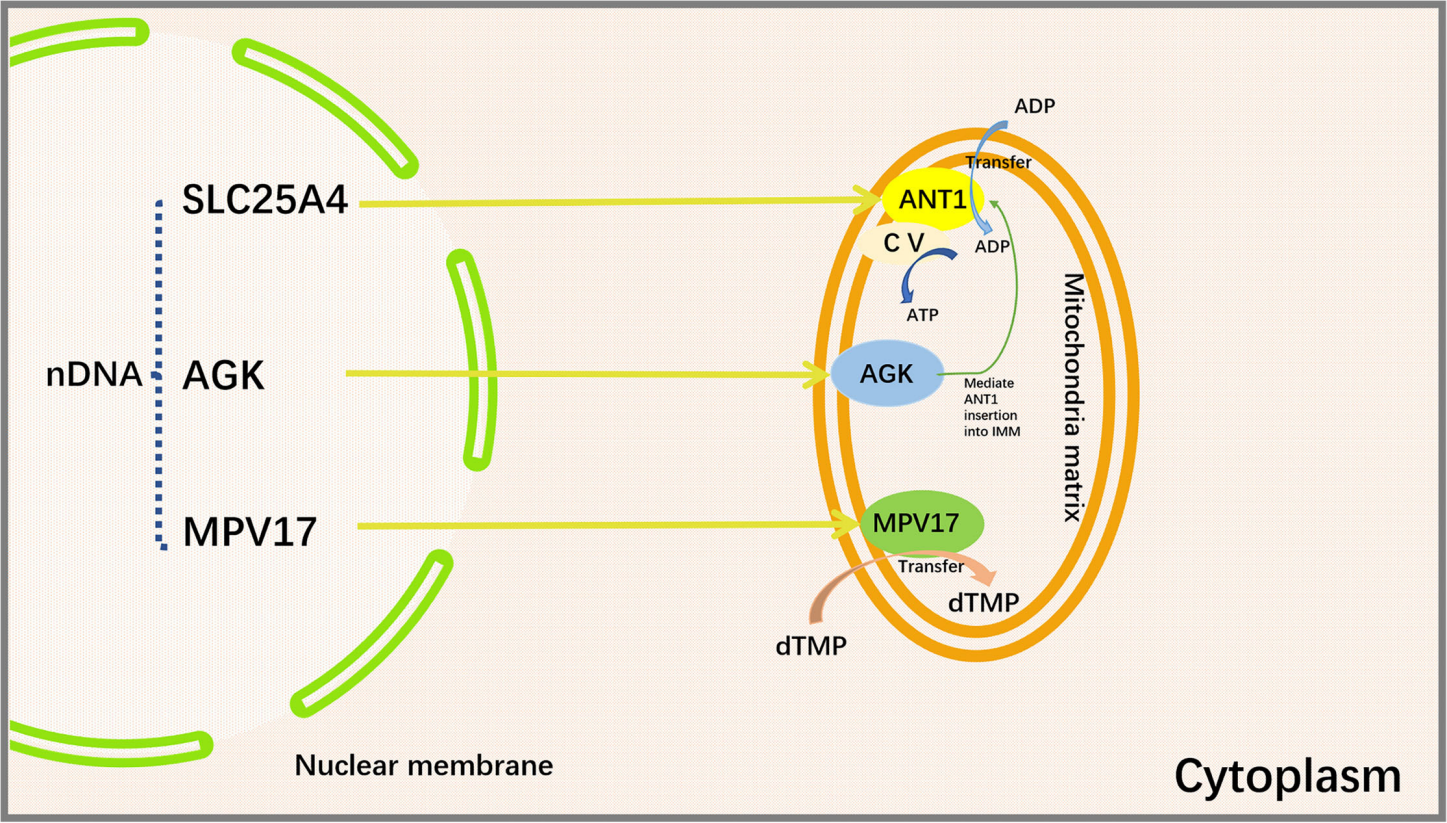
Proteins encoded by *SLC25A4, AGK*, and *MPV17* genes and their functions. *SLC25A4, AGK* and MPV are important mitochondrial transport proteins. Adenine nucleotide transporter (ANT1) is an ADP/ATP translocator encoded by *SLC25A4* and located on the inner mitochondrial membrane. It mediates the transmembrane exchange of ADP and ATP and provides ADP for mitochondrial ATP synthase. Acylglycerol kinase is a mitochondrial lipid kinase encoded by *AGK* gene. The absence of *AGK* reduces the content of ANT1 in the inner membrane of muscle mitochondria by affecting lipid metabolism. *MPV17* protein is encoded by *MPV17* gene, which is involved in the maintenance of nucleotide homeostasis and transfers dTMP from the cytoplasm to the mitochondria to maintain mtDNA synthesis. *SLC25A4*, Solute Carrier Family 25 Member 4; *AGK*, Acylglycerol Kinase; *MPV17*, Mitochondrial Inner Membrane Protein *MPV17*; ANT1, Adenine nucleotide translocase 1; CV, Complex V, also known as ATP synthase, is one of the mitochondrial respiratory chain electron transport complexes; ADP, Adenosine diphosphate; ATP, Adenosine triphosphate; dTMP, deoxythymidine monophosphate.

*MPV17* is a nuclear-encoded IMM protein expressed in the brain, kidneys, liver, spleen, and heart in humans (115). A large number of studies have demonstrated that the deficiency of *MPV17* may cause abnormalities in mitochondrial ROS production flux, ETC complex activity, mitochondrial membrane potential, and pH value (116, 117). Judith R. Alonzo showed that *Mpv17* could transfer dTMP from cytoplasm to mitochondria for mtDNA synthesis (118). *Mpv17* deficiency may cause a depletion of dTMP pool in the mitochondria (119). Loss of *MPV17* protein leads to a notable reduction in liver mtDNA and ultimately liver dysfunction (120). Mutations in *MPV17* gene can result in neurological problems and failure to thrive in pediatric patients (121). The absence of *MPV17* protein does not appear to significantly affect mtDNA quantity and overall function of the heart in normal adult mice. However, cardiac mitochondria from *MPV17* mutant mice displayed severe cristae damage after ischemia/reperfusion (I/R) injury. *MPV17* mutation also disrupted its interactions with ATP synthase, MICOS, Cyclophilin D, and glucose-regulated protein 75 (GRP75). Mitochondrial sensitivity to Ca^2+^ overload was enhanced in I/R mice, thereby promoting mPTP opening and leading to subsequent cardiomyocyte death (117) (Figure 3).

#### POLG

*POLG* is the only DNA polymerase γ (POLγ) in the mitochondria. This enzyme is a heterologous trimer consisting of three subunits, which are encoded by *POLG* and *POLG2* genes on chromosome 15. *POLG* gene is a common pathogenic gene of MDS, in which hundreds of mutations have been identified (122). When this gene is mutated, mitochrondria lose the ability to maintain the genome, leading to mtDNA depletion. A large number of mutation sites in *POLG* have been reported, including the homozygous mutation Chr17:62492543 G>A of Chr17 located in *POLG2* gene, which leads to the replacement of R182W in P55. The clinical manifestations of the patient include the reduction in mtDNA content and liver failure, indicative of hepatocerebral MDS. In addition, Hoff et al. showed that the Chr17:62492543 G>A mutation in *POLG*2 and R182W p55 severely damaged the stability of the auxiliary subunit, and was associated with the disease phenotype (123). Recent studies identified a new homozygous mutation [c.2391G>T (p.M797I)] in the C-terminal subdomain of *POLG* in a close family with MNGIE syndrome (124). Heart-specific overexpression of POLγY955C mutant caused dysregulation mtDNA biogenesis, resulting in cardiac dysfunction and eventually premature death. Utilizing transgenic mice expressing human POLγY955C in the heart, William Lewis et al. demonstrated that the mice had defective enzyme machinery for mtDNA replication, which resulted in the phenotype of mtDNA depletion. In addition, the mice also showed the phenotypes of increased mitochondrial oxidative stress, histopathological changes, and abnormal mitochondrial ultrastructure in cardiomyocytes. Moreover, cardiac insufficiency is accompanied by cardiac enlargement, increased ventricular volume, and heart mass (125).

#### RRM2B

*RRM2B* (ribonucleotide reductase regulatory TP53 inducible subunit M2B), is located on chromosome 8q22.3, and encodes for ribonucleotide diphosphate reductase subunit M2B and p53-inducible ribonucleotide reductase (RNR) small subunit (p53R2). RNR is responsible for catalyzing free radicals and reducing ribonucleotides, in order to dNTPs to maintain the dNTP library at an appropriate level for DNA synthesis and repair. When this gene is defective, it often leads to the occurrence of mitochondrial diseases. Changes in the structural properties of *RRM2B* protein can also cause mtDNA damage and affect the nervous system (126, 127). Stojanović et al. has reported a missense mutation c.707G>A, p.Cys236Tyr in *RRM2B* gene in the blood cells of a white male. This mutation appears to be associated with congenital deafness and exercise intolerance due to the impairment of nerve conduction (128). Mutations in *RRM2B* gene also cause MNGIE. Two *RRM2B* pathogenic mutations, namely, c.362G>A (p.R121H) and c.329G>A (p.R110H), were identified to be associated with severe mtDNA depletion in a 42-year-old woman. The mutations changed the docking interface of ribonucleoside reductase (RIR2B) homodimeric, thereby affecting the activity of the enzyme (118). In addition, *RRM2B* mutations can also cause cardiovascular disease. In the case report by Albert Z Lim et al. defects in mtDNA maintenance were found in patients with left ventricular hypertrophy, cardiomyopathy and ventricular septal defects (129).

#### TWNK

Twinkle (*TWNK*, previously designated as C10orf2), is responsible for encoding Twinkle proteins, maintaining mtDNA integrity, and displaying 5′ → 3′ DNA helicase activity when unwinding mitochondrial DNA. Mutations in this gene are associated with mitochondrial DNA depletion syndrome 7 (MTDPS7). Li et al. found that the proband carried two *TWNK* compound heterozygous mutations, of which c.1186C>T (p.Pro396Ser) in exon 1 was inherited from the father, and c.1844G>C (p.Gly615Ala) in exon 5 was inherited from the mother (130). Dominant mutations in *TWNK* cause progressive ophthalmoplegia, mtDNA deletion, and autosomal dominant inheritance 3, while recessive mutations often lead to MTDPS 7 and Perrault syndrome 5. Among them, MTDPS7 is usually manifested by infantile-onset spinocerebellar ataxia (IOSCA). In a report by Kume et al. *TWNK* homozygous mutations could lead to cerebellar ataxia in middle-aged patients (131, 132). Twinkle protein has three main domains: primary enzyme domain, junctional domain, and helical enzyme domain. The entire primary enzyme and connector domains as well as a partial helix enzyme domain are located in exon 1. Exons 2–4 encode the remaining helix enzyme domain. Kume et al. (131) found the amino acid at codon 292 changed from Pro to Thr in IOSCA patients. In addition to homozygous mutations in the helix domain, homozygous mutations in the primary enzyme domain 9 and compound heterozygous mutations in both primary enzyme and helix domain 10 were reported in IOSCA patients (133). Pohjoismäki et al. showed that Twinkle protein encoded by *TWNK* gene may play a direct role in the maintenance of human heart (133). The copy number of myocardial mitochondrial DNA decreased when heart failure occurred, especially after myocardial infarction. Overexpression of Twinkle helicase could greatly increase the copy number of mtDNA, thereby protecting the heart (134). In addition, the overexpression of *TWNK* also decreased the accumulation of ROS-induced mtDNA mutations, thus benefiting cardiomyopathy in Sod2 +/– mice (135).

#### FBXL4

*FBXL4* (F-box and leucine-rich repeat protein 4), located on chromosome 6, is a nDNA that encodes 621-amino-acid F-box protein. *FBXL4* plays vital roles in maintaining mtDNA integrity and stability. It is the first protein family member reported to form Skp1-Cullin-F-box (SCF) (136). In the fibroblasts of patients with *FBXL4* double allele mutations, the dysregulated mitochondrial network, decreased mitochondrial energy metabolism-related enzyme activity and reduced consumption of mtDNA were observed. The mutations can lead to mitochondrial exhaustion syndrome 13 (MTDPS13) of cerebral muscular disease type, which is often manifested as decreased muscle tone, difficulty in eating, delayed nerve development, brain atrophy and other symptoms, accompanied by increased blood lactic acid level (137, 138). Wang et al. found a novel *FBXL4* frameshift mutation (c.993dupA) in a Han Chinese patient, leading to the premature termination of protein synthesis and defects at the protein level (110). Ballout et al. reported the first case of MTDPS13 in Lebanon. Full-exon group sequencing showed that the female patient had a homozygous non-sense mutation c. 1303C>T (p.Arg435^*^). In addition to the typical clinical manifestations such as encephalopathy and muscle tone reduction, anemia and normal blood ammonia also appeared (139). Emperador et al. reported new *FBXL4* mutations in two patients, which were composite heterozygous mutations c.[858 + 5G>C]; [1510T>C] and homozygous mutation c.[851delC]; [851delC]. Besides, skeletal muscle biopsy showed severe mtDNA depletion (140). In a case reported by Huemer et al. (137) cardiac disease was diagnosed in seven patients with *FBXL4* mutations at an initial visit. Tissue examination and cell staining indicated joint defects in the respiratory chain, reduced production of enzymes related to mitochondrial energy metabolism, and reduced mitochondrial DNA content (136). Antoun et al. reported that *FBXL4* deficiency could result in severe multi-system diseases, including lactic acidosis, cystic white matter disease, cardiomyopathy, arrhythmia, and immunodeficiency (141). A patient with prenatal diagnosis of polyhydramnios and cerebellar atrophy showed mitochondrial encephalomyopathy. This patient suffered from myocardial hypertrophy with ventricular septal defect. Seven of the nine patients (77%) with *FBXL4* deficiency showed abnormal cardiac manifestations, including fallot syndrome (TOF), supraventricular tachycardia (SVT), ventricular septal defect (ASD), and left or right ventricular hypertrophy. These findings imply that cardiac involvement may be one of the most common clinical features of *FBXL4* deficiency (142). It has been reported that two siblings harbor homozygous variants in *FBXL4* gene (c.1750 T>C; p.Cys584Arg). They had symptoms of encephalomyopathy, lactic acidosis and myocardial hypertrophy, which were consistent with the characteristics of mitochondrial injury myopathy caused by *FBXL4* gene mutation. Dichloroacetic acid (DCA) treatment effectively alleviated metabolic acidosis and reversed myocardial hypertrophy in the younger sister (143).

#### TFAM

The mitochondrial transcription factor A (*TFAM*) gene, located on nuclear chromosome 10q21, is composed of six introns and seven exons. *TFAM* is a member of the HMG box protein family. It contains two HMG box domains, an N-terminal domain and a C-terminal structure. *TFAM* has the ability to maintain mtDNA and initiate mtDNA transcription (144, 145). *TFAM* is a key factor in promoter selection during the initiation of mitochondrial transcription sites. It recognizes the promoter of *POLRMT* and initiates transcription by binding to mtDNA without sequence specificity (146). Stile et al. idenfied a *TFAM* homozygous missense variant (c.533C>T; p. Pro178Leu) among the close relatives of Basque lineage in Colombia. Two siblings showed IUGR, elevated transaminase, preterm infant with hypoglycemia and hyperbilirubinemia progressed to liver failure and death (147). In addition, *TFAM* and mtDNA copy numbers are essential for the control of mitochondrial gene expression; their reduction can lead to poor heart function and aging (148, 149). Heart-specific knockout of *TFAM* induced apoptosis in the heart of a transgenic mouse model. Disruption of *TFAM* in the heart resulted in mtDNA depletion, reduced mitochondrial ATP production rate, and impaired respiratory chain enzyme activities. These defects aggravate heart disease and eventually lead to heart failure (146, 150).

#### MGME1

Mitochondrial Genome Maintenance Exonuclease 1 (*MGME1* or *DDK1*) belongs to PD-(D/E)XK nuclease superfamily. It is a highly conserved mitochondrial-specific DNA enzyme encoded by *C20orf72 gene*. Mutation of *C20orf72* is associated with exhaustion and rearrangement of mtDNA. Patients with *C20orf72* mutations may have CPEO, myasthenia, respiratory distress and gastrointestinal diseases (151). Mouse studies by Matic et al. suggest that *MGME1* gene product may be a part of the regulatory switch at the end of the D-loop region since it regulates the replication of mtDNA and the termination of H-chain transcription. Lack of *MGME1* may lead to the deletion or depletion of mtDNA in mouse tissue cells (152). *MGME1* may also contribute to cerebellar ataxia since mutation of this gene leads to the occurrence of mtDNA depletion syndrome 11, with a series of clinical manifestations of cerebellar diseases. Recently, Hebbar et al. identified a new code-shift mutation c.359del (p.Pro120Leufs^*^2) in exon 2 in a child with early progressive cerebellar ataxia. The investigator determined that the mutation was the cause of MTDPS11 (153). A high-throughput mutation screening of 16 patients with dilated or hypertrophic cardiomyopathy indicated that *MGME1* variants are highly associated with the pathogenesis of heart disease (154).

#### QIL1/MIC13(C19orf70)

*MICOS13*, also known as *C19orf70, MIC13*, or *QIL1* (155, 156), is a component of mitochondrial contact site and cristae tissue system (MICOS) complex. It plays vital roles in maintaining the characteristics of mitochondrial cristae and cellular energy metabolism. *MICOS13* deficiency leads to morphological defects of cristae and cristae junctions and severely impairs the IMM architecture and mitochondrial respiratory function (157). Patients from a consanguineous family were reported to have *MICOS* p.(Gly15Glufs^*^75) variant, thus resulting in mitochondrial hepato-encephalopathy. Homozygous individuals displayed syndromes correlated with mitochondrial dysfunction. In addition, multiple organs with high energy demands, including heart, liver and the central nervous system, were damaged (155, 158). It has been reported that two siblings with *QIL1* deficiency exhibited mitochondrial encephalopathy symptom. Loss of *QIL1* in this case showed abnormalities in multiple organs (e.g., liver and muscle), while heart ultrasound indicated mild heart hypertrophy as early as 2 years old (159). Furthermore, *QIL1* point mutation also resulted in cardiac arrhythmia but not cardiac hypertrophy (160).

## Summary and Outlook

MDS is a mitochondrial disease characterized by the reduced amount and/or depletion of mtDNA content. They are caused by mutations in nDNA and can involve two or more organs. In this paper, while discussing the impact of different types of MDS on muscle, brain and nervous system, we explored the potential association between different gene mutations and MDS-associated cardiac diseases, in order to provide guidance for early screening and prevention of these diseases.

## Author Contributions

JY and YX: conceptualization and resources. HW, YX, and JY: original draft preparation and resources. YH, SL, YuC, YaC, JingW, and YuZ: writing. YH, SL, YuC, YaC, YuZ, YaZ, JingsW, HW, and JY: review and editing. All authors have read and agreed to the published version of the manuscript.

## Funding

The authors would like to acknowledge funding from the Research Start-up Fund of Jining Medical University (Reference: 600791001), the Foundation of Medical Health Science and Technology Development Program of Shandong Province (Reference: 202002061311), the Research Support Fund for Teachers of Jining Medical University (Reference: JYFC2019KJ013), and the Jining Medical College Student Innovation and Entrepreneurship Training Program Project (Reference: cx2021053).

## Conflict of Interest

The authors declare that the research was conducted in the absence of any commercial or financial relationships that could be construed as a potential conflict of interest.

## Publisher's Note

All claims expressed in this article are solely those of the authors and do not necessarily represent those of their affiliated organizations, or those of the publisher, the editors and the reviewers. Any product that may be evaluated in this article, or claim that may be made by its manufacturer, is not guaranteed or endorsed by the publisher.

## Figures and Tables

**Table:** 

**Full Name**	**Abbreviations**
Outer Mitochondrial Membrane	OMM
Inner Mitochondrial membrane	IMM
Mitochondrialintermembrane space	IMS
Mitochondrial Respiratory Chain	MRC
Respiratory Chain	RC
Mitochondrial Permeability Transition Pore	MPTP
Mitochondria lDNA	mtDNA
nuclear DNA	nDNA
Ribosomal RNA	rRNA
transfer RNA	tRNA
Mitochondrial DNA depletion syndrome	MDS
Oxidative Phosphorylation	OXPHOS
Electron Transport Chain	ETC
Ubiquinone oxidoreductase subunit AB1	NDUFAB1
cytochrome C	Cyt c
ADP/ATP carrier	AAC
apoptosis-inducing factor	AIF
coenzyme Q10	CoQ10
supercomplex	SCs
Mitochondrial Membrane Potential	MMP
Reactive Oxygen Species	ROS
Nicotinamide Adenine Dinucleotide	NADH
acyl carrier protein	ACP
Tricarboxylic Acid Cycle	TCA
reduced flavin adenosine dinucleotide	FADH2
hypoxia inducible factor	HIF
Prolyl Hydroxylase	PHD
Hypoxia Response Element	HRE
Fatty Acid β-Oxidation	FAO
acetyl Coenzyme A	acetyl-CoA
nicotinamide adenine dinucleotide	NAD+
flavin adenine dinucleotide	FAD
AMP-activated protein kinase	AMPK
mitochondrial protein	Mfn
dynein-related protein-like 1	Drp1 / DLP1
optic nerve atrophy 1	OPA1
Pyruvate Dehydrogen-ase Phosphatase 1	PDP1
Pyruvate Decarboxylase	PDH
α-Ketoglutarate Dehydrogenase	α-KGDH
mitochondrial permeability transition pore	MPTP
Sarcoplasmic Reticulum	SR
type 2 Ryanodine receptor	RyR2
Mitochondrial Calcium Uniporter	MCU
Mitochondrial Calcium Uptake 1	MICU1
Endoplasmic Reticulum	ER
Mitchondria Associated Endoplasmic Reticulum Membrane	MAM
Mitochondrial-Derived Peptide	MDP
mitochondrial DNA depletion syndrome-2	MTDPS2
Creatine Kinase	CK
Thymidine Kinase 2	TK2
Deoxythymidine	dThd
deoxycytidine monophosphate	dCMP
deoxythymidine monophosphate	dTMP
external eye muscle paralysis	adPEO
Mitochondrial Depletion Syndrome 13	MTDPS 13
Mitochondrial Depletion Syndrome 9	MTDPS 9
Cytochrome-C Oxidase	COX
Mitochondrial Neurogastrointestinal Encephalopathy Disease	MNGIE
Thymidine Phosphorylase	TP
vascular endothelial growth factor	VEGF
Thymidine	Thd
Deoxyuridine	dUrd
deoxyuridine triphosphate pool	dNTP
Chronic Progressive External Ophthalmoplegia	CPEO
oxidized low density protein	oxLDL
heart failure	HF
cardiovascular disease	CVD
thymidylate synthase	TS
Thymidine Triphosphate	TTP
Thymidine monophosphate	TMP
Polymerase Gamma	POLG
DNA Polymeraseγ	POLγ
Succinate COA ligases	SUCL
Succinate-CoA Ligase GDP/ADP-Forming Subunit ?	SUCLG 1
Ribonucleotide Reductase Regulatory TP53 Inducible Subunit M2B	RRM2B
Ribonucleotide Reductase	RNR
Ribonucleoside Reductase	RIR2B
Mitochondrial Depletion Syndrome 7	MTDPS 7
Infantile-onset Spinocerebellar Ataxia	IOSCA
Adenine Nucleotide Translocase	ANT1
mitochondrial exhaustion syndrome 12	MTDPS12
Skp1-Cullin-F-box	SCF
Dichloroacetic acid	DCA
mitochondrial transcription factor A	TFAM
mitochondrial contact site and cristae tissue system	MICOS
Mitochondrial Contact Site And Cristae Organizing System Subunit 13	MICOS13
box and leucine-rich repeat protein 4	FBXL4
Twinkle	TWNK
Solute Carrier Family 25 Member 4	SLC25A4
Mitochondrial Inner Membrane Protein MPV17	MPV17
mitochondrial exhaustion syndrome 13	MTDPS13
fallot syndrome	TOF
ventricular septal defect	ASD
supraventricular tachycardia	SVT
glucose-regulated protein 75	GRP75
Mitochondrial Genome Maintenance Exonuclease 1	MGME1
Acylglycerol Kinase	AGK
